# High-Throughput CRISPR Screens To Dissect Macrophage-*Shigella* Interactions

**DOI:** 10.1128/mBio.02158-21

**Published:** 2021-12-21

**Authors:** Yong Lai, Liang Cui, Gregory H. Babunovic, Sarah M. Fortune, John G. Doench, Timothy K. Lu

**Affiliations:** a Antimicrobial Resistance Interdisciplinary Research Group, Singapore-MIT Alliance for Research and Technology, Singapore; b Synthetic Biology Group, MIT Synthetic Biology Center, Massachusetts Institute of Technology (MIT), Cambridge, Massachusetts, USA; c Research Laboratory of Electronics, MIT, Cambridge, Massachusetts, USA; d Department of Immunology and Infectious Diseases, Harvard T. H. Chan School of Public Health, Boston, Massachusetts, USA; e Ragon Institute of MGH, MIT, and Harvard, Cambridge, Massachusetts, USA; f Broad Institute, Cambridge, Massachusetts, USA; g Department of Electrical Engineering and Computer Science, MIT, Cambridge, Massachusetts, USA; h Harvard-MIT Division of Health Sciences and Technology, Cambridge, Massachusetts, USA; i Department of Biological Engineering, MIT, Cambridge, Massachusetts, USA; Korea Advanced Institute of Science and Technology

**Keywords:** host-pathogen interactions, shigellosis, host-directed therapy, CRISPR-Cas9 knockout screen, CRISPRi screen, macrophages, TLR1/2 signaling, pyruvate catabolism, genome-wide CRISPR screens

## Abstract

Shigellosis causes most diarrheal deaths worldwide, particularly affecting children. *Shigella* invades and replicates in the epithelium of the large intestine, eliciting inflammation and tissue destruction. To understand how *Shigella* rewires macrophages prior to epithelium invasion, we performed genome-wide and focused secondary CRISPR knockout and CRISPR interference (CRISPRi) screens in Shigella flexneri-infected human monocytic THP-1 cells. Knockdown of the Toll-like receptor 1/2 signaling pathway significantly reduced proinflammatory cytokine and chemokine production, enhanced host cell survival, and controlled intracellular pathogen growth. Knockdown of the enzymatic component of the mitochondrial pyruvate dehydrogenase complex enhanced THP-1 cell survival. Small-molecule inhibitors blocking key components of these pathways had similar effects; these were validated with human monocyte-derived macrophages, which closely mimic the *in vivo* physiological state of macrophages postinfection. High-throughput CRISPR screens can elucidate how S. flexneri triggers inflammation and redirects host pyruvate catabolism for energy acquisition before killing macrophages, pointing to new shigellosis therapies.

## INTRODUCTION

Shigellosis is an infectious disease caused by one of four species of *Shigella* bacteria: Shigella flexneri, Shigella sonnei, Shigella dysenteriae, and Shigella boydii (1). S. flexneri causes 65% of *Shigella* infections globally, especially in low- and middle-income countries (1). Annually, there are more than 1 million cases of shigellosis (1). In 2016, over 200,000 people were killed by shigellosis globally (2). Although shigellosis is a self-limiting disease, the World Health Organization (WHO) 2005 guidelines recommend antimicrobial treatment to prevent complications and reduce diarrheal deaths (3). However, resistance to first-line treatment, fluoroquinolones, is widespread because of the frequency of the triple mutations *gyrA* S83L and D87G and *parC* S80I in the chromosome of *Shigella* strains (4, 5). Additionally, plasmid-mediated resistance to second-line treatments, such as ampicillin, azithromycin, and cefixime, raises additional concerns for the acquisition of more antibiotic resistance genes by horizontal gene transfer in *Shigella* strains (6, 7). The WHO and the Centers for Disease Control and Prevention (CDC) have declared antibiotic-resistant *Shigella* to be a serious public health threat that requires novel approaches for therapy (8, 9). More than 65% of shigellosis deaths occurred in children under 5 years of age and in adults older than 70 years (2), indicating that in children and middle-aged adults, the fully developed, healthy human immune system may be sufficient to prevent and control *Shigella* infections. We therefore focused on immune cells to investigate susceptibility to this potentially lethal bacterium at the cellular level.

Infection is initiated when *Shigella* crosses the intestinal epithelium through microfold cells (M cells) (10). After transcytosis to the M cell pocket, *Shigella* targets the resident macrophages and dendritic cells. As it multiplies within macrophages, *Shigella* induces caspase-1-dependent pyroptotic cell death, a step essential to subsequent invasion and replication in the intestinal epithelium (11, 12). Epithelial cells constitute the major habitat of *Shigella* (13). Within this replicative niche, Shigella flexneri delivers various virulence proteins via a type III secretion system (T3SS), resulting in weakened host defenses (13, 14). These virulence proteins reduce intracellular trafficking (15, 16), antagonize caspase-4-dependent pyroptosis (17), prevent necrosis mediated by mitochondrial damage (18), and inhibit the early stage of apoptosis by p53 degradation (19). As a consequence, epithelial cells survive infection and continue to harbor the bacteria (19–21). Infected epithelial cells secrete chemokines and cytokines, which recruit macrophages, natural killer cells, and polymorphonuclear leukocytes to the sites of infection (13). Additionally, *Shigella* invades and manipulates cells of the adaptive immune system, including T and B cells, by T3SS effectors (13).

Whereas the effects of *Shigella* infection on epithelial cells have been studied extensively, little attention has been paid to how S. flexneri interacts with macrophages. Yet understanding these interactions is crucial to redirecting the immune response to shield against this bacterial infection. What we know so far is that this intracellular pathogen rapidly induces macrophage pyroptosis by activating inflammasomes, specifically NLRP3 (NLR family pyrin domain-containing protein 3) and NLRC4 (NLR family CARD domain-containing protein 4) (22–24). So far, two genome-wide screens have been conducted to systematically investigate complex host-pathogen interactions in S. flexneri infection: an RNA interference (RNAi) screen in epithelial HeLa cells (25) and a loss-of-function screen in human-derived haploid (Hap-1) cells (26). To improve our understanding of how S. flexneri manipulates macrophages and induces rapid cell death and to identify host targets for potential therapy, we employed CRISPR screens, which are highly efficient and have minimal off-target effects. We first screened human macrophage-like THP-1 cells infected with S. flexneri and then validated the function of small-molecule inhibitors in a primary human monocyte-derived macrophage (hMDM) infection model.

## RESULTS

### Host cell survival-based genome-wide primary screens.

To simplify the analysis of intracellular pathogen infection and facilitate comparison between the previously published genome-wide RNAi screen in epithelial cells (25) and our CRISPR screens in human monocytic THP-1 cells, we used the same bacterial strain: the S. flexneri M90T Δ*virG* mutant (S. flexneri Δ*virG*), which has lost the capability of cell-to-cell spread. To examine whether this mutant strain invades and kills THP-1 cells, we first assessed its phagocytosis by detecting the expression of a reporter. The reporter is a red fluorescent protein (RFP) in S. flexneri whose expression is driven by the native promoter of the S. flexneri *uhpT* gene (P*uhpT*::dsRed) (27). Reporter expression is specifically induced by host cell-produced glucose 6-phosphate (27) and indicative of intracellular S. flexneri (see Fig. S1A in the supplemental material). The fluorescent reporter is activated only when the bacteria are present in the cytosol following their entry into the host cells. THP-1 cell viability postinfection was determined by trypan blue staining (Fig. S1B to S1E). We found that S. flexneri Δ*virG*, at a multiplicity of infection (MOI) of 10:1, efficiently infected undifferentiated THP-1 cells and induced host cell death 3 h after infection, which is similar to the results of previously published reports of the effects of S. flexneri in THP-1 cells (2-h infection at an MOI of 50), Caco-2 cells (2-h infection at an MOI of 1 or 100), and organoid-derived cell monolayers (3-h incubation) (Fig. S1D and S1E) (24, 28, 29). This result indicated that infection with S. flexneri Δ*virG* could be utilized as selective pressure for subsequent host survival-based genetic screens.

10.1128/mBio.02158-21.1 FIG S1 Optimization of conditions for *Shigella* infection of THP-1 cells. (A) S. flexneri Δ*virG* (P*uhpT*::dsRed) infection at an MOI (multiplicity of infection, or the number of bacterial cells per host cell) of 10, from 0.5 h to 5 h of incubation with undifferentiated THP-1 cells (scale bar, 20 μm). (B to E) The number of viable THP-1 cells after 0.5 to 5 h of infection was determined by trypan blue staining. (D) More than 90% of host cells were killed after 3 h of infection. N.D, not detectable. Data represent means ± SD (*n* = 3) (Student’s two-tailed unpaired *t* test, *, *P* < 0.05; **, *P* < 0.01; ***, *P* < 0.001; ns, not significant). Download FIG S1, TIF file, 1.6 MB. Copyright © 2021 Lai et al. 2021 Lai et al. https://creativecommons.org/licenses/by/4.0/ This content is distributed under the terms of the Creative Commons Attribution 4.0 International license.

**FIG 1:**
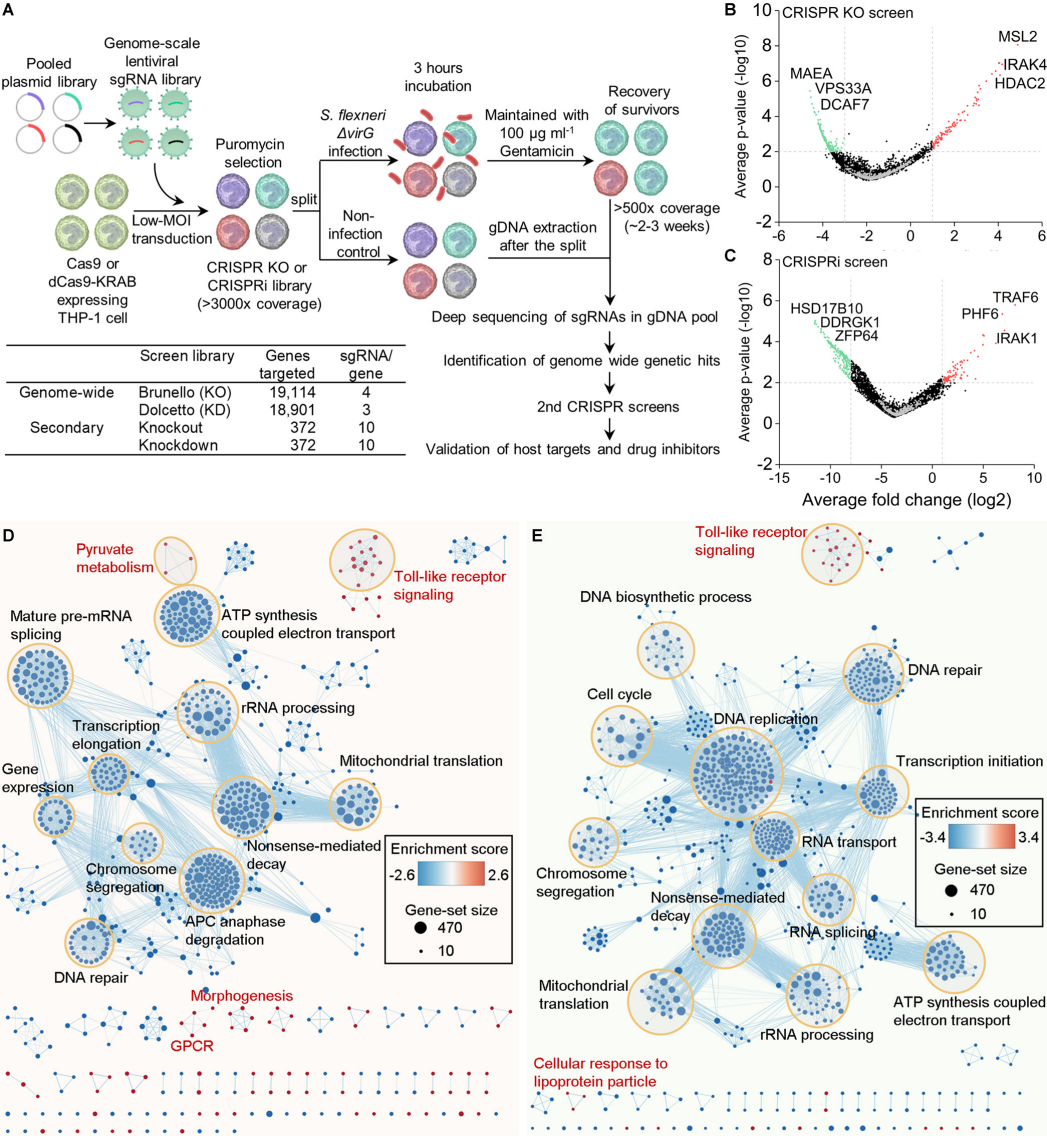
Genome-wide pooled CRISPR knockout and CRISPRi screens to dissect biological pathways in S. flexneri infection. (A) Monoclonal Cas9 and dCas9-Krab-expressing THP-1 cell lines were constructed and transduced with lentiviral sgRNA libraries. High-coverage CRISPR knockout and CRISPRi libraries were split for subsequent S. flexneri Δ*virG* infection. Uninfected host cells were collected for genomic DNA extraction immediately after the split. Surviving THP-1 cells with sgRNA barcodes were harvested until the total number reached >500-fold sgRNA coverage of screen libraries (2 to 3 weeks) and processed for next-generation sequencing. Genome-wide genetic hits were identified by comparing sgRNA abundances between infected samples and noninfected controls. Based on those host targets, secondary CRISPR knockout and CRISPRi screen libraries were designed and prepared. Similarly, host cell survival-based secondary positive screens were performed to validate those host targets. Finally, drug inhibitors that selectively inhibit genetic hits were tested. KO, knockout; KD, knockdown. (B and C) Volcano plots from genome-wide CRISPR knockout (B) and CRISPRi (C) screens. For each sgRNA-targeted gene, the *x* axis shows its enrichment (positive hits) or depletion (negative hits) postinfection, and the *y* axis shows statistical significance measured by *P* value. The top 3 positive and negative screen hits are labeled as red and green dots, respectively; positive hits were those that extended the survival of the THP-1 cells beyond 2 to 3 h of bacterial infection and negative hits those that shortened THP-1 cell survival. Gray dots represent nontargeting controls. For each screen, experiments were carried out in triplicate. (D and E) Genes identified by genome-wide CRISPR knockout (D) and CRISPRi (E) screens were functionally categorized to understand the biological functions involved in S. flexneri infection. The color gradient of nodes represents the enrichment scores of gene sets. Node size represents the number of genes in the gene set.

Independent biological triplicates of genome-wide CRISPR knockout and CRISPR interference (CRISPRi) screen libraries were prepared in THP-1 cells expressing Cas9 and dCas9-Krab, respectively (Fig. 1A) (30–32). After 3 h of S. flexneri Δ*virG* infection, surviving THP-1 cells with specific single guide RNA (sgRNA) barcodes were maintained in culture medium with gentamicin for continuous replication (∼2 to 3 weeks) until the total number of surviving cells reached 500-fold sgRNA coverage. Cells were then harvested for next-generation sequencing (NGS) and analysis. The distribution of sgRNAs in S. flexneri Δ*virG*-infected THP-1 cells was significantly different from that in uninfected THP-1 cells (Fig. S2A to S2F). The results of genome-wide screens were visualized with volcano plots (Fig. 1B and C and Fig. S2G to S2L). Pathway analysis identified both depleted and enriched biological processes in S. flexneri Δ*virG*-infected THP-1 cells (Fig. 1D and E). Most of these biological pathways, many of which are essential for host cell functions, were depleted postinfection. Yet, CRISPR-Cas9 knockout and CRISPRi screens also identified several enriched biological pathways, such as the Toll-like receptor (TLR) signaling and pyruvate metabolism pathways.

10.1128/mBio.02158-21.2 FIG S2 sgRNA distribution and genetic hits in genome-wide CRISPR knockout and CRISPRi screening. (A to C) Distribution of individual sgRNAs in control and S. flexneri Δ*virG*-infected samples after CRISPR knockout screens. (D to F) Distribution of individual sgRNAs in control and S. flexneri Δ*virG*-infected samples after CRISPRi screens. Each point represents an individual sgRNA. Boxes, 25th to 75th percentiles; whiskers, 1st to 99th percentiles. (G to I) Volcano plots of results from independent biological triplicates from CRISPR knockout screens. (J to L) Volcano plots of results from independent biological triplicates from CRISPRi screens. For each sgRNA-targeted gene, the *x* axis shows its enrichment (positive hits) or depletion (negative hits) postinfection, and the *y* axis shows statistical significance measured by *P* value. The top 5 positive and negative screen hits are labeled as red and blue dots, respectively. Download FIG S2, TIF file, 2.5 MB. Copyright © 2021 Lai et al. 2021 Lai et al. https://creativecommons.org/licenses/by/4.0/ This content is distributed under the terms of the Creative Commons Attribution 4.0 International license.

**FIG 2:**
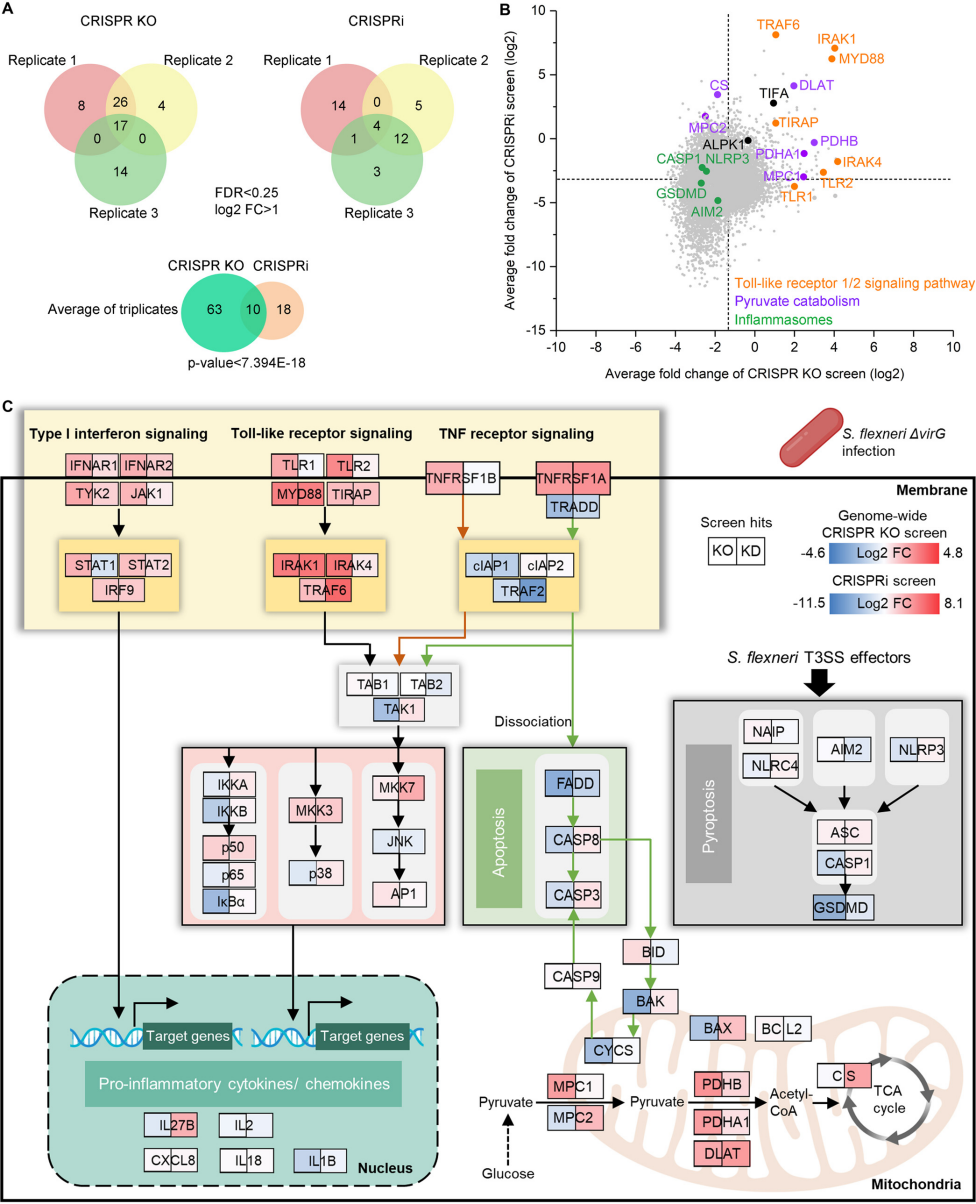
Genome-wide CRISPR knockout and CRISPRi screens to dissect enriched genes and biological pathways in S. flexneri infection. (A) Enriched genes in the Venn diagram were filtered with a cutoff FDR of <0.25 and log_2_ fold change of >1 in S. flexneri Δ*virG* infection. The degree of significance of the overlap between genome-wide CRISPR knockout and CRISPRi screens is given. (B) Gene-centric visualization of the average log_2_ fold change of CRISPR knockout and CRISPRi screens in S. flexneri-infected versus noninfected host cells. Selected components of TLR1/2, the pyruvate catabolism signaling pathway, and inflammasome formation are highlighted in orange, purple, and green, respectively. (C) Top enriched genes and associated biological pathways in S. flexneri Δ*virG* infection. The color gradient of gene boxes represents the log_2_ fold change of gene sets in genome-wide CRISPR knockout and CRISPRi screens. TCA, tricarboxylic acid.

In order to identify top positively selected genetic hits in S. flexneri Δ*virG*-infected THP-1 cells, we used a false-discovery rate (FDR) of <0.25 and log_2_ fold change (FC) of >1 as cutoff points. Positive hits were considered those that extended the survival of the THP-1 cells beyond 2 to 3 h of S. flexneri Δ*virG* infection. The CRISPR knockout screen, done in triplicate, identified more enriched genetic hits than the CRISPRi screens (Fig. 2A). We observed positive selection of 73 and 28 genes in CRISPR-Cas9 knockout and CRISPRi screens, respectively, with 10 genes enriched in both screens (*P* value of overlap, <7.394E–18) (Fig. 2A). Pathway analysis identified multiple enriched biological processes in S. flexneri Δ*virG*-infected THP-1 cells. Both CRISPR-Cas9 knockout and CRISPRi screens identified the same pathways, such as TLR cascades, pathways involved in chromatin organization and pyruvate metabolism, the cellular stress response pathway, and receptor tyrosine kinase signaling (Fig. S3A and S3B; see also Tables S1 and S2 at https://doi.org/10.17632/xn3vv2cdnk.1). Specifically, all key components of the TLR1/2 signaling pathway were identified as positive hits in our genome-wide screens. These were TRAF6 (TNF receptor-associated factor 6), IRAK1 (interleukin 1 receptor-associated kinase 1), IRAK4 (interleukin 1 receptor-associated kinase 4), MYD88 (myeloid differentiation primary response 88), TLR1, TLR2, and TIRAP (TIR domain-containing adaptor protein) (Fig. 2B and C; Tables S3 and S4 at https://doi.org/10.17632/xn3vv2cdnk.1). Importantly, TRAF6, TIFA (TRAF-interacting protein with forkhead-associated domain), and TLR1 are top genetic hits identified by a previously published genome-wide RNAi screen in HeLa cells, which supported the reliability of our CRISPR screens in THP-1 cells (Fig. S4) (25). Intriguingly, TRAF6 and TIFA also play important roles in the ALPK1 (alpha kinase 1)–TIFA–TRAF6–NF-κB pathway, by which epithelial cells detect lipopolysaccharide (LPS) biosynthetic intermediates and regulate inflammation in response to them (25, 33, 34). Yet, ALPK1, a cytosolic immune receptor identified in Yersinia pseudotuberculosis and S. flexneri infection in epithelial cell lines (33, 35), was not a genetic hit in our genome-wide CRISPR screens, possibly because this receptor has different functions in epithelial cells and macrophages during intracellular pathogen infection (Fig. 2B). Moreover, several components of the type I interferon (IFN) and the tumor necrosis factor (TNF) receptor signaling pathways, which induce proinflammatory cytokine and chemokine production or activate apoptotic cell death, were identified as positive hits; these included IFNAR1 (interferon alpha and beta receptor subunit 2), IFNAR2, STAT1 (signal transducer and activator of transcription 1), STAT2, IRF9 (interferon regulatory factor 9), TNFRSF1A (tumor necrosis factor receptor superfamily member 1A), TNFRSF1B, and two hits previously identified by the genome-wide RNAi screen in HeLa cells noted above, i.e., TYK2 (tyrosine kinase 2) and JAK1 (Janus kinase 1) (Fig. 2C and Fig. S4) (25). Although S. flexneri Δ*virG* causes NF-κB-induced inflammation in both macrophages and epithelial cells, it also exhibits distinct mechanisms of host manipulation that may contribute to opposite outcomes of infection in these cell types, i.e., rapidly induced cell death of macrophages but inhibited cell death of epithelial cells (13).

10.1128/mBio.02158-21.3 FIG S3 Enrichment of biological pathways identified by genome-wide CRISPR knockout and CRISPRi screens in S. flexneri infection of THP-1 cells. (A and B) Gene enrichment analysis of positive genetic hits identified by a CRISPR knockout screen (A) and CRISPRi screen (B) in S. flexneri Δ*virG* infection (FDR < 0.1, log_2_ FC > 1). Download FIG S3, TIF file, 1.4 MB. Copyright © 2021 Lai et al. 2021 Lai et al. https://creativecommons.org/licenses/by/4.0/ This content is distributed under the terms of the Creative Commons Attribution 4.0 International license.

10.1128/mBio.02158-21.4 FIG S4 Comparison of five screens: the two genome-wide CRISPR screens described in this study, an RNAi screen, and a loss-of-function screen in which S. flexneri infected various host cells, plus a CRISPR screen in which a pathogen-associated molecular pattern (PAMP) was substituted for bacterial infection. A genome-wide CRISPR knockout (Brunello) screen, CRISPRi (Dolcetto), RNAi screens, a loss-of-function screen in haploid cells, and CRISPR knockout (GeCKO v2) screens are labeled in blue, red, green, yellow, and brown, respectively. Download FIG S4, TIF file, 0.5 MB. Copyright © 2021 Lai et al. 2021 Lai et al. https://creativecommons.org/licenses/by/4.0/ This content is distributed under the terms of the Creative Commons Attribution 4.0 International license.

Moreover, the identification of genes as positive hits involved in pyruvate catabolism in macrophages, such as PDHB (pyruvate dehydrogenase E1 subunit beta), DLAT (dihydrolipoamide *S*-acetyltransferase), CS (citrate synthase), PDHA1 (pyruvate dehydrogenase E1 subunit alpha 1), MPC1 (mitochondrial pyruvate carrier 1), and MPC2 (Fig. 2B and C), echoed the rerouting of carbon flux by another S. flexneri strain with a similar knockout mutation, the S. flexneri 2457T Δ*virG* mutant, observed in epithelial HeLa cells (36). The altered carbon flux supports the rapid growth of intracellular bacteria using pyruvate as a carbon source in the host cell (37).

Intriguingly, in our genome-wide CRISPR screens, knockout or knockdown of key components of the NLRC4 and NLRP3 inflammasomes, i.e., AIM2 (absent in melanoma 2), NLRP3, CASP1 (caspase 1), and GSDMD (gasdermin D), did not enhance host cell survival, although those host genes have been shown to be targets of the S. flexneri T3SS effectors MxiI (22), IpaB (38), and IpaH7.8 (39) in macrophages. In contrast, the survival of cells with knockout or knockdown of AIM2, NLRP3, CASP1, or GSDMD was slightly shortened in S. flexneri Δ*virG* infection, indicating that these genes in the pyroptotic cell death pathway actually may not be detrimental to host cells infected with S. flexneri (Fig. 2B and C; see also Table S5 at https://doi.org/10.17632/xn3vv2cdnk.1).

Overall, the identification of known host targets and the same pathways by both CRISPR knockout and CRISPRi screens indicates the reliability of genome-wide CRISPR screens for studying S. flexneri infection. In addition, the identification of previously unknown host targets demonstrates the importance of comprehensively understanding macrophage-S. flexneri interactions.

### Focused secondary screens for host-pathogen interactions.

**FIG 3:**
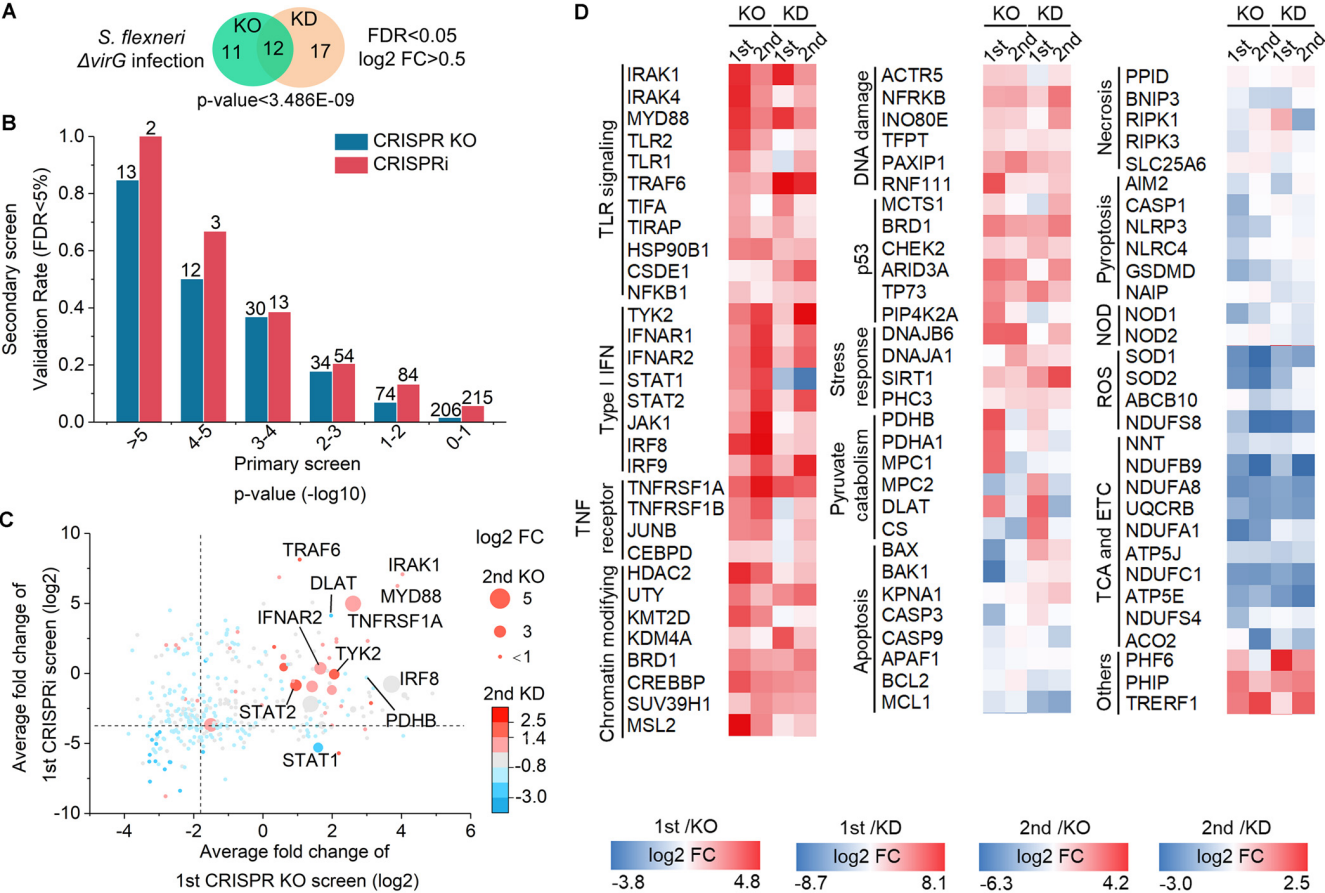
Secondary CRISPR knockout and CRISPRi screens identify host genetic hits in S. flexneri infection. (A) Enriched genes were filtered with a cutoff FDR of <0.05 and a log_2_ fold change of >0.5 in S. flexneri Δ*virG* infection. The degree of significance of the overlap is given. (B) Validation rates of genetic hits in the secondary screen grouped by their *P* values in the genome-wide screens in S. flexneri Δ*virG* infection. The number of genes per category is indicated. (C) Genetic hits from both primary genome-wide and secondary screens were ranked by their differential sgRNA abundances between S. flexneri-infected and uninfected populations (log_2_ fold change). (D) Heatmap of screen hits clustered in different biological pathways in S. flexneri Δ*virG* infection. ETC, electron transport chain; ROS, reactive oxygen species; NOD, nucleotide binding oligomerization domain.

We next designed and prepared secondary CRISPR knockout and CRISPRi screen libraries targeting 372 human genes (31), in order to validate the genome-wide screen hits. The secondary-screen libraries were also used to test genes that were associated with different types of host cell death and to compare the performance of CRISPR knockout libraries with that of CRISPR knockdown libraries by ensuring that there were consistent numbers of sgRNAs per gene in each type of library (10 sgRNAs per gene) (Fig. 1A). As in the procedure used in our genome-wide screens, surviving THP-1 cells were harvested following S. flexneri Δ*virG* infection, and the results of the screens were visualized with volcano plots (Fig. S5A and S5B). We identified 23 and 29 genes in secondary CRISPR knockout and CRISPRi screens, respectively; 12 genes were enriched in both screens (FDR < 0.05, log_2_ FC > 0.5, *P* value < 3.486E–09) (Fig. 3A). To evaluate the reliability of our genome-wide CRISPR screens, we calculated the validation rates of genes in secondary screens based on the FDR threshold (<5%); these genes were clustered by their *P* value in the genome-wide screens (Fig. 3B) (32). The validation rate of screen hits in secondary CRISPR knockout and CRISPRi screens decreased with increasing *P* values in primary genome-wide screens. This result suggests the reliability of genome-wide screens, which have fewer sgRNAs per gene than secondary-screen libraries, for studying bacterial infections (Fig. 1A). Furthermore, top genome-wide positive genetic hits, such as genes in the TLR1/2 signaling pathway (IRAK1, MYD88, TRAF6), the type I interferon pathway (TYK2, IFNAR2, IRF8, STAT2), and the TNF receptor signaling pathway (TNFRSF1A), were validated by secondary screens, suggesting the robustness of genome-wide screens (Fig. 3C and Fig. S5C; Tables S6 and S7 at https://doi.org/10.17632/xn3vv2cdnk.1). Yet, genes involved in pyruvate catabolism, such as PDHB and PDHA1, were not scored significantly in secondary screens (Fig. S5C); additional validation of these genes would be required to confirm their involvement in the infectious process. Positive screen hits that were identified by both genome-wide and secondary screens were clustered in heat maps based on the diverse signaling pathways with which they are associated during S. flexneri Δ*virG* infection (Fig. 3D). The advantage of using genome-wide screens to comprehensively identify host targets was indicated by the identification of many positive genetic hits with unknown functions in bacterial infection, such as PHF6 (PHD finger protein 6), PHIP (pleckstrin homology domain-interacting protein), and TRERF1 (transcriptional regulating factor 1) (Fig. 3D and Tables S3 and S4 at https://doi.org/10.17632/xn3vv2cdnk.1).

10.1128/mBio.02158-21.5 FIG S5 Genetic hits identified by secondary CRISPR screens in *Shigella* infection of THP-1 cells. (A and B) Volcano plots from secondary CRISPR knockout (A) and CRISPRi (B) screens. For each sgRNA-targeted gene, the *x* axis shows its enrichment (positive hits) or depletion (negative hits) postinfection, and the *y* axis shows statistical significance measured by *P* value. The top 3 positive and negative screen hits are labeled as red and green dots, respectively. Gray dots represent nontargeting controls. For each screen, experiments were carried out in triplicate. (C) Gene-centric visualization of average log_2_ fold changes for secondary screens in S. flexneri-infected versus noninfected host cells. Selected components of the TLR1/2 signaling pathway, pyruvate catabolism, inflammasome formation, and the type I interferon signaling pathway are highlighted in orange, purple, green, and pink, respectively. Download FIG S5, TIF file, 1.2 MB. Copyright © 2021 Lai et al. 2021 Lai et al. https://creativecommons.org/licenses/by/4.0/ This content is distributed under the terms of the Creative Commons Attribution 4.0 International license.

Consistently with our genome-wide CRISPR screens, host cell survival was shortened in our secondary screens by knockout or knockdown of components of inflammasomes mediating pyroptosis in macrophages postinfection, such as GSDMD and NLRP3 (Fig. 3D and Fig. S5C). These genes were not identified as either positive or negative hits in the genome-wide screens because of their high FDRs; however, the secondary screens revealed them to be negative hits (Table S5 at https://doi.org/10.17632/xn3vv2cdnk.1). Genes involved in other types of host cell death, i.e., necrosis and apoptosis, did not show consistent patterns of screen phenotypes and were not identified as genetic hits. Not surprisingly, NOD1 (nucleotide binding oligomerization domain containing 1), a critical intracellular bacterial sensor, was identified as a negative hit in the CRISPR knockout screen, indicating its normally protective role for host cells (Fig. 3D and Fig. S5C). Intriguingly, SOD1 (superoxide dismutase 1) and SOD2, which destroy free superoxide radicals in host cells, were also identified as negative screen hits in S. flexneri Δ*virG* infections (Fig. 3D and Fig. S5C); decreased expression of these genes may contribute to necrosis induced by reactive oxygen species (40). Additionally, by calculating the sgRNA correlation of replicates, signal/noise ratio, *P* value, and FDR of CRISPR screens, we found that CRISPR-Cas9 knockout and CRISPRi yielded comparable results in secondary screens (Fig. S6).

10.1128/mBio.02158-21.6 FIG S6 Comparison of secondary CRISPR knockout and CRISPRi screens in S. flexneri infection of THP-1 cells. (A to D) sgRNA-level correlation of replicates in CRISPR knockout screens (A and B) and CRISPRi screens (C and D). The Pearson correlation of log_2_ fold change values between replicates is indicated. (E and F) Top 50 positive genetic hits (red) and nontargeting controls (gray) identified by CRISPR knockout (E) and CRISPRi (F) screens in S. flexneri Δ*virG* infection. Broken lines show the means of the hits (*M*_signal_) and nontargeting controls (*M*_bckg_). (G and H) *P* value (G) and FDR (H) of the top 100 positive genetic hits identified by CRISPR knockout and CRISPRi screens in S. flexneri Δ*virG* infection. Download FIG S6, TIF file, 2.5 MB. Copyright © 2021 Lai et al. 2021 Lai et al. https://creativecommons.org/licenses/by/4.0/ This content is distributed under the terms of the Creative Commons Attribution 4.0 International license.

### Validation of screen hits for host-pathogen interactions.

**FIG 4:**
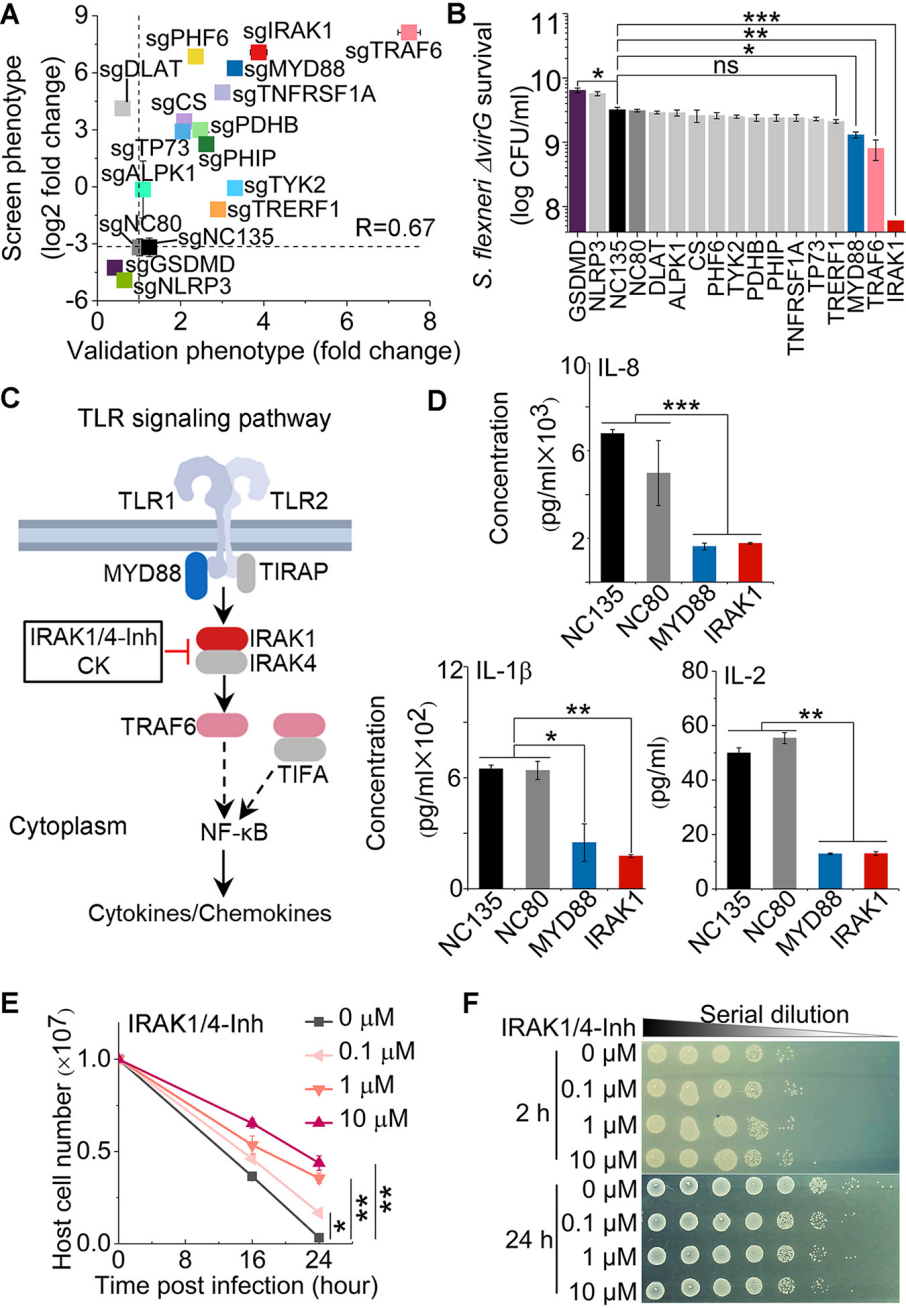
Validation of top genetic hits and effects of the IRAK1 inhibitor in S. flexneri infection of human THP-1 cells. (A) Correlation between pooled screen and validation data. For each hit, the log_2_ fold change obtained from the genome-wide CRISPRi screening data (screen phenotype) was plotted against the fold change of cell viability of genetic hits from levels in the nontargeting control cells (validation phenotype). Host cell viability was measured by trypan blue staining. sgNC80 and sgNC135 are nontargeting controls. *R* is the Pearson correlation coefficient. (B) Intracellular S. flexneri Δ*virG* level after infection of individual knockdown THP-1 cells, which was measured by counting bacterial CFU. (C) Schematic of positive genetic hits in the TLR1/2 signaling pathway and corresponding inhibitors. (D) Cytokine and chemokine production in infected THP-1 cells with MYD88 and IRAK1 knockdown. (E and F) Viability of THP-1 cells (E) and growth of the intracellular S. flexneri Δ*virG* mutant (F) postinfection in the presence or absence of the IRAK1 inhibitor IRAK1/4-Inh at different concentrations. Data represent the means ± standard deviations (SD) (*n* = 3) (Student's two-tailed unpaired *t* test, *, *P* < 0.05; **, *P* < 0.01; ***, *P* < 0.001; ns, not significant).

To verify the function of the top positive and negative genetic hits, we next constructed THP-1 cells with individual gene knockdowns using the most efficient sgRNAs identified in our genome-wide CRISPRi screens. These experiments allowed us to confirm the corresponding cellular phenotypes of S. flexneri Δ*virG* infection. The genes that we investigated were associated with the TLR1/2 signaling pathway (IRAK1, MYD88, and TRAF6), pyruvate catabolism (PDHB, DLAT, and CS), the type I interferon pathway (TYK2), the TNF receptor signaling pathway (TNFRSF1A), a cytosolic immune receptor (ALPK1), p53 regulation (TP73), and inflammasomes (GSDMD and NLRP3), as well as unknown functions in bacterial infection (PHF6, PHIP, and TRERF1). The positive correlation between screen phenotype and validation phenotype confirmed that repression of positive screen hits indeed enhanced host cell survival, with a 93.3% true-positivity rate (Pearson *R* = 0.67) (Fig. 4A). Moreover, repression of the transcription of MYD88, TRAF6, and IRAK1, key components in the TLR1/2 signaling pathway, also inhibited the intracellular growth of S. flexneri Δ*virG*, as measured by counting bacterial CFU (Fig. 4B). In contrast, repression of the transcription of GSDMD and NLRP3 indeed decreased host cell survival (GSDMD *P* value = 0.0038; NLRP3 *P* value = 0.0185), and GSDMD gene knockdown increased intracellular pathogen growth in S. flexneri Δ*virG* infection (Fig. 4A and B).

To characterize how the inhibition of those positive genetic hits mediates the host cell response and provides protection, we measured cytokine and chemokine production regulated by the TLR1/2 signaling pathway (Fig. 4C). Knockdown of the transcription of either MYD88 or IRAK1 abolished the production of infection-induced proinflammatory cytokines and chemokines, such as interleukin 8 (IL-8), IL-1ꞵ, and IL-2 (Fig. 4D and Fig. S7A). As a potential strategy to control intracellular bacterial infection by targeting host factors, we tested the functions of corresponding small-molecule inhibitors IRAK1/4-inhibitor I (IRAK1/4-Inh) and ginsenoside compound K (CK). IRAK1/4-Inh, a selective inhibitor of IRAK1, inhibited proinflammatory cytokine and chemokine production and protected host cells in a dose-dependent manner, as seen by determining viability with trypan blue staining (Fig. 4E and Fig. S7B to S7D). Unlike the similar levels of intracellular S. flexneri Δ*virG* after 2 h of infection, the number of intracellular pathogens decreased in the presence of IRAK1/4-Inh 24 h postinfection, indicating that inhibition occurred by controlling intracellular pathogen growth rather than by blocking pathogen entry (Fig. 4F). CK, a metabolite of Panax ginseng that also inhibits IRAK1 (41), similarly enhanced host cell survival, inhibited the intracellular growth of S. flexneri Δ*virG*, and abolished infection-induced IL-8 production in THP-1 cells (Fig. S8).

10.1128/mBio.02158-21.7 FIG S7 Cytokine and chemokine production in THP-1 cells in S. flexneri infection. (A) Cytokine and chemokine production in THP-1 cells with or without S. flexneri Δ*virG* infection. (B to D) Production of IL-1ꞵ (B), IL-2 (C), and IL-8 (D) in THP-1 cells in the presence of the IRAK1 inhibitor at different concentrations. Data represent the means ± SD (*n* = 3) (Student’s two-tailed unpaired *t* test, *, *P* < 0.05; **, *P* < 0.01; ***, *P* < 0.001; ns, not significant). Download FIG S7, TIF file, 0.7 MB. Copyright © 2021 Lai et al. 2021 Lai et al. https://creativecommons.org/licenses/by/4.0/ This content is distributed under the terms of the Creative Commons Attribution 4.0 International license.

10.1128/mBio.02158-21.8 FIG S8 Validation of IRAK1 inhibitor compound K in *Shigella* infection. (A) Survival of THP-1 cells postinfection in the presence or absence of different concentrations of compound K (CK). Viability was determined by trypan blue viable cell staining. (B) Intracellular growth of S. flexneri Δ*virG* in the presence or absence of different concentrations of CK. (C) Production of infection-induced IL-8 in the presence or absence of different concentrations of CK. (D) Growth of THP-1 cells in the presence or absence of CK without S. flexneri infection. Data represent the means ± SD (*n* =3) (Student’s two-tailed unpaired *t* test, *, *P* < 0.05; **, *P* < 0.01; ns, not significant). Download FIG S8, TIF file, 1.2 MB. Copyright © 2021 Lai et al. 2021 Lai et al. https://creativecommons.org/licenses/by/4.0/ This content is distributed under the terms of the Creative Commons Attribution 4.0 International license.

**FIG 5:**
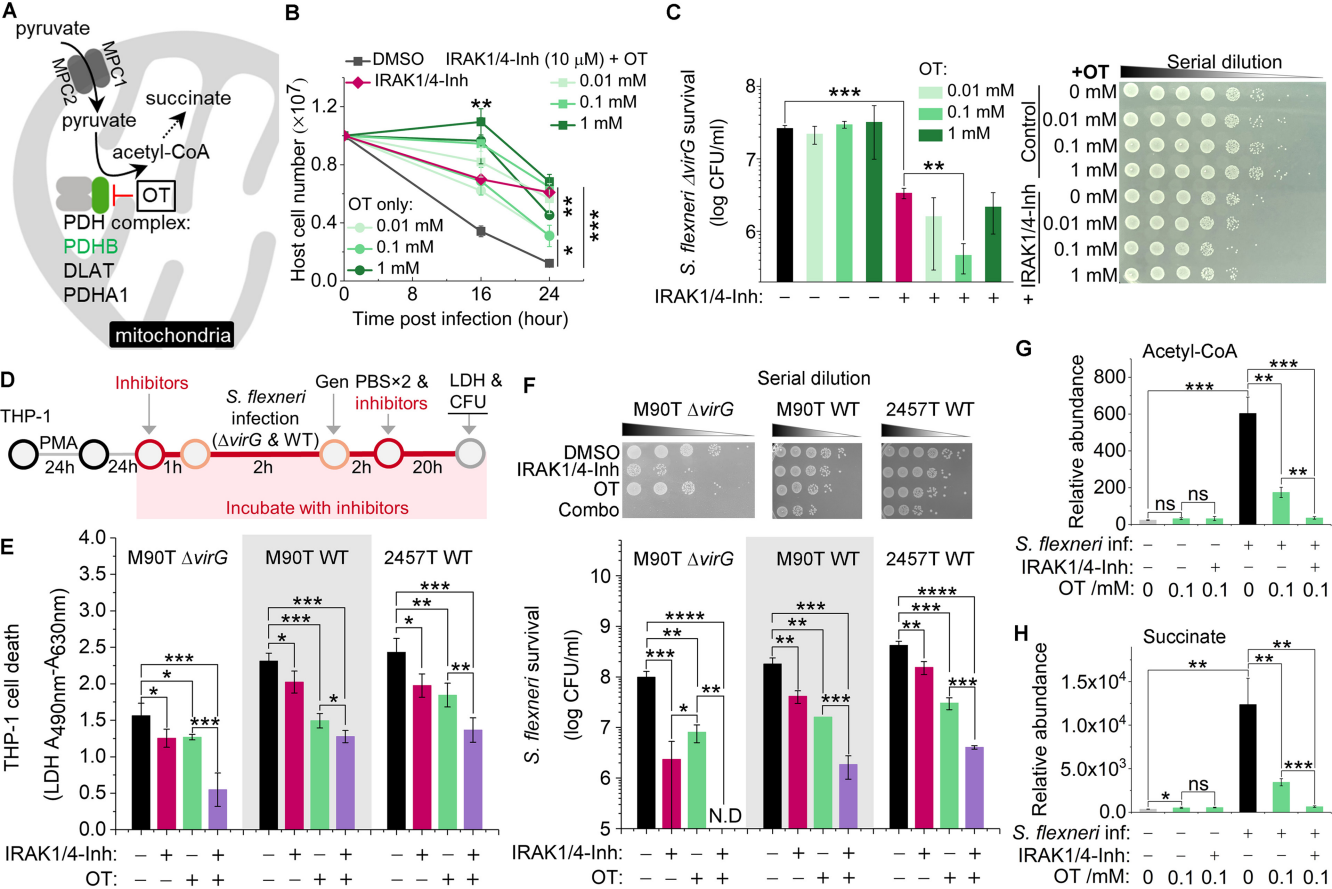
Validation of positive genetic hits in the pyruvate catabolism signaling pathway and effects of the corresponding inhibitor in S. flexneri infection of human THP-1 cells. (A) Schematic of positive genetic hits in the pyruvate catabolism signaling pathway and the corresponding inhibitor. (B and C) Growth of THP-1 cells (B) and the intracellular level of S. flexneri Δ*virG* (C) postinfection in the presence or absence of various concentrations of the PDHB inhibitor (oxythiamine, OT) or OT combined with IRAK1/4-Inh (10 μM). (D) Schematic of inhibitor validation in PMA-stimulated THP-1 cells infected with different S. flexneri strains. Gen, gentamicin. (E) Effects of IRAK1 and PDHB inhibitors on the survival of differentiated THP-1 cells after infection with S. flexneri M90T Δ*virG* (M90T Δ*virG*), the S. flexneri M90T wild type (M90T WT), and the S. flexneri 2457T wild type (2457T WT), with death measured by LDH release. (F) Effects of the IRAK1 and PDHB inhibitors on the growth of three intracellular S. flexneri strains in differentiated THP-1 cells, as measured by CFU. IRAK1/4-Inh was used at 10 μM. OT was used at 0.1 mM. (G and H) Production of acetyl-CoA (G) and succinate (H) with or without S. flexneri Δ*virG* infection and in the presence or absence of OT or OT combined with the IRAK1 inhibitor (10 μM). Data represent the means ± SD (B, C, G, and H, *n* = 3; E and F, *n* = 4) (Student's two-tailed unpaired *t* test, *, *P* < 0.05; **, *P* < 0.01; ***, *P* < 0.001; ****, *P* < 0.0001; ns, not significant; N.D, not detectable).

In addition to dysregulating the host immune response, S. flexneri grows rapidly and replicates in host cells but does so only if there is an adequate supply of nutrients. The survival of THP-1 cells infected with S. flexneri Δ*virG* was also favored by knockout or knockdown of components of the pyruvate dehydrogenase complex or the pyruvate transporter MPC1/2 in the mitochondrion-redirected central metabolism (Fig. 3D and 5A). These results are congruent with the induction by S. flexneri Δ*virG* in epithelial cells of the production of acetyl coenzyme A (acetyl-CoA) (36). We next tested the function of the PDHB inhibitor oxythiamine (OT), as well as its combination with IRAK1/4-Inh, in S. flexneri Δ*virG* infection. OT treatment enhanced host cell survival postinfection, as measured by trypan blue staining (Fig. 5B), but failed to control intracellular S. flexneri Δ*virG* growth, as measured by counting bacterial CFU (Fig. 5C), which is consistent with the PDHB gene knockdown phenotype (Fig. 4A and B). Interestingly, the combination of both inhibitors (IRAK1/4-Inh and OT) significantly enhanced host cell survival and controlled the growth of S. flexneri Δ*virG* better than treatment with either of these inhibitors alone, indicating a synergistic effect of inhibitors targeting both immune and nonimmune pathways in macrophages (Fig. 5B and C).

To ensure that host cells continued to proliferate and to enable cell survival-based genetic screening, we used undifferentiated THP-1 cells in CRISPR screens and host target validation. We next tested the function of inhibitors in phorbol myristate acetate (PMA)-stimulated THP-1 S. flexneri infection models (Fig. 5D). Consistently with results for undifferentiated THP-1 cells, IRAK1/4-Inh, OT, and their combination enhanced host cell survival, as measured by lactate dehydrogenase (LDH) release, and limited the intracellular growth of S. flexneri Δ*virG* (Fig. 5E and F). Considering that the *virG* gene affects host cell adhesion and actin-based motility, to exclude the effects of attenuated S. flexneri Δ*virG* in infection, we tested the functions of small-molecule inhibitors in THP-1 cells infected with either of two wild-type S. flexneri strains (Fig. 5D) (42–44). Indeed, more host cell death was induced by wild-type S. flexneri M90T or wild-type S. flexneri 2457T than by S. flexneri Δ*virG*, and higher levels of intracellular pathogen growth were observed for these wild-type strains (Fig. 5E and F). Moreover, IRAK1/4-Inh, OT, and their combination each significantly reduced host cell death and intracellular wild-type pathogen survival, indicating that the effects of these small-molecule inhibitors was not restricted to infection with the mutant strain S. flexneri Δ*virG* (Fig. 5E and F).

In line with a previous study of epithelial cells (36), we found that S. flexneri Δ*virG* induced acetyl-CoA production in THP-1 cells, suggesting that, in both cases, S. flexneri Δ*virG* supports its own rapid intracellular growth and replication by manipulating the central metabolism of the host cell (Fig. 5G). Moreover, a 0.1 mM concentration of the PDHB inhibitor (OT) decreased infection-induced acetyl-CoA and downstream succinate production, which shifts host metabolism and leads to enhanced host cell survival (Fig. 5G and H). The combination of both the IRAK1 and PDHB inhibitors reduced acetyl-CoA and succinate production to uninfected-cell levels, thus limiting intracellular S. flexneri growth and propagation (Fig. 5G and H).

**FIG 6:**
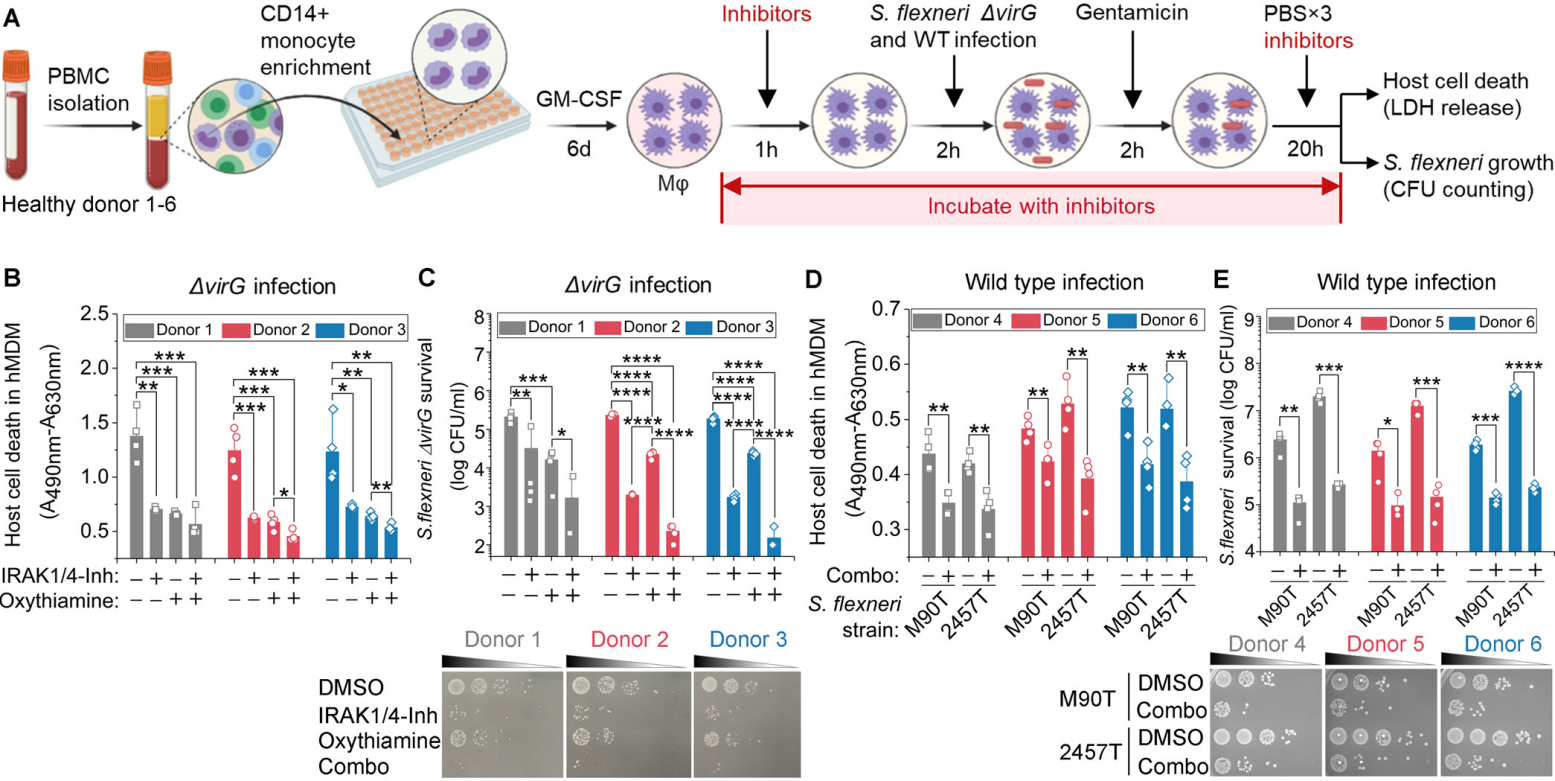
Validation of inhibitors in S. flexneri infection of primary human macrophages (Mφ). (A) Schematic of inhibitor validation in S. flexneri Δ*virG* infection cell models. (B) Effects of IRAK1 and PDHB inhibitors on the survival of primary human monocyte-derived macrophages (hMDMs) infected with S. flexneri, with host cell death measured by LDH release. (C) Effects of IRAK1 and PDHB inhibitors on the growth of intracellular S. flexneri Δ*virG* in hMDMs, as measured by CFU. (D) Effects of the combination of IRAK1 and PDHB inhibitors (Combo) on the survival of hMDMs infected with one of two S. flexneri wild-type strains, S. flexneri M90T and S. flexneri 2457T, which were grown in TSB before infection. (E) Effects of the combination of IRAK1 and PDHB inhibitors on the growth of intracellular S. flexneri wild-type strains in hMDMs, as measured by CFU. IRAK1/4-Inh was used at 10 μM. Oxythiamine (OT) was used at 0.1 mM. Data represent the means ± SD (*n* = 4) (Student's two-tailed unpaired *t* test, *, *P* < 0.05; **, *P* < 0.01; ***, *P* < 0.001; ****, *P* < 0.0001). 6d, 6 days.

To further validate the function of these inhibitors, we tested host cell death and intracellular S. flexneri growth in a primary human macrophage infection model (Fig. 6A). Consistently with the results of the THP-1 infection model, IRAK1/4-Inh, OT, and their combination each significantly reduced the death of hMDMs by intracellular S. flexneri Δ*virG*, as measured by LDH release (Fig. 6B). Those small-molecule inhibitors also restricted the intracellular growth of S. flexneri Δ*virG* in these cells, as measured by counting bacterial CFU (Fig. 6C). To exclude any effects of the bacterial growth medium, two wild-type S. flexneri strains, M90T and 2457T, were cultivated in tryptic soy broth (TSB) prior to the infection of hMDMs. The combination of IRAK1/4-Inh and OT enhanced hMDM survival and controlled the intracellular growth of both of the wild-type S. flexneri strains under these conditions (Fig. 6D and E). Thus, inhibiting the TLR1/2 signaling pathway or the pyruvate catabolism signaling pathway restricts the intracellular pathogen burden and preserves the survival of human macrophages infected with S. flexneri.

## DISCUSSION

Our study highlights the ability of host cell survival-based CRISPR screens to elucidate complex macrophage-pathogen interactions and to identify key cellular processes and gene products that are disrupted by intracellular pathogens. It thus reinforces the value of CRISPR screens for understanding pathogenesis (45, 46). Our pooled screen is more cost-effective and technologically feasible than plate-based large-scale genetic screens for identifying genetic hits without requiring consideration of the indirect impact on cells in the whole population. Because we included nontargeted control host cells in our CRISPR screen libraries and compared sgRNA abundances with and without infection, this screen was especially effective for identifying genes whose function is affected by *Shigella* infection. Also highlighted is the importance of screening gene perturbations in the specific cell types that are infected by an intracellular pathogen, especially in the instance of intracellular bacteria, such as *Shigella*, that invade and inhabit more than one type of human cell.

Rapid macrophage death is prerequisite for S. flexneri to infect and persist in epithelial cells, which ultimately results in diarrhea and even dysentery, the most life-threatening manifestations of infection. However, unlike with the intensive studies of the effects of *Shigella* infection on epithelial cells, the comprehensive interactions between S. flexneri and macrophages have been largely overlooked (22–24, 38, 39). In epithelial cells, NF-κB-related inflammatory signaling is one of the major defenses against S. flexneri infection (25, 33). For instance, upon sensing ADP-β-d-manno-heptose (ADP-Hep), epithelial cells activate NF-κB signaling in their cytosol and produce the proinflammatory chemokine IL-8 (33). In response, S. flexneri produces multiple virulence proteins, disrupting inflammation and preventing epithelial cell death (14). However, what occurs in epithelial cells does not necessarily occur in other cell types. In fact, our study has revealed effects of S. flexneri in macrophage-like THP-1 cells and primary human macrophages that are distinct from the effects reported for *Shigella*-infected epithelial cells.

In THP-1 cells, S. flexneri also stimulated IL-8 production but did so by activating the TLR1/2 signaling pathway, and infection induced rapid THP-1 cell death. Although the TLR1/2 pathway is well known for its role in the innate immune response to invading pathogens via the recognition of peptidoglycan and triacyl lipopeptides, the induced inflammation may contribute to bacterial pathogenesis (47). For instance, in *Burkholderia* infection, knockout of TLR2 enhances the survival of mice and reduces sepsis, compared to what was observed with wild-type mice (48). Moreover, the blockade of both TLR2 and TLR4 with monoclonal antibodies effectively inhibits the immunopathology triggered by Escherichia coli and Salmonella enterica and prevents mouse death (49). In our study, inhibiting the TLR1/2 signaling pathway in THP-1 cells by IRAK1 inhibitors reduced proinflammatory cytokine and chemokine production, enhanced the survival of both THP-1 cells and primary human macrophages, and limited intracellular S. flexneri growth and replication, indicating the detrimental effect of the TLR1/2 signaling pathway on innate immune cells during S. flexneri infection. Furthermore, we found that host cell targets of S. flexneri virulence factors that had been identified in epithelial cells were not identified by our screens as positive genetic hits in THP-1 cells (see Table S8 in the supplemental material). Considering the opposite effects of NF-κB signaling and inflammation when either epithelial cells or immune cells are infected with S. flexneri, modulating the inflammatory response of the host as a therapeutic strategy should be very carefully considered, and future efforts should be made to conduct CRISPR screens in human intestinal organoids with both immune and epithelial cells.

10.1128/mBio.02158-21.9 TABLE S8 Average log_2_ fold change of host cell targets of S. flexneri virulence factors in genome-wide CRISPR screens. Download Table S8, DOCX file, 0.02 MB. Copyright © 2021 Lai et al. 2021 Lai et al. https://creativecommons.org/licenses/by/4.0/ This content is distributed under the terms of the Creative Commons Attribution 4.0 International license.

Compared to TLR1/2 signaling and pyruvate catabolic pathways, caspase-1-induced pyroptosis did not play a major role in our infection model. It is well known that pyroptosis is triggered by S. flexneri, allowing bacteria to escape from macrophages and invade epithelial cells (13). However, this inflammatory programmed cell death also mediates immunity to infection and controls a variety of bacterial pathogens, including S. flexneri, in animal models (50, 51). Pyroptosis restricts intracellular bacterial pathogens by cytokine-independent mechanisms in a mouse model as an efficient innate immune response (52) or by the direct antibacterial effect of GSDMD-NT (the N-terminal cleavage product of GSDMD involved in pyroptosis), which binds to cardiolipin and forms pores on the membranes of both Gram-positive and Gram-negative bacteria (53, 54). It has been unclear whether pyroptosis protects the macrophages or the intracellular bacteria in *Shigella* infection. Our host genetic perturbation strategies provide direct causal evidence that some of the genes that contribute to pyroptosis actually benefit the host cells, since knockout or knockdown of key components of pyroptosis (GSDMD and NLRP3) decreased host cell survival postinfection (Fig. 3D and 4A; Table S5 at https://doi.org/10.17632/xn3vv2cdnk.1) and increased intracellular pathogen growth (GSDMD) (Fig. 4B).

Treatment for shigellosis is becoming increasingly difficult as resistance to most inexpensive and widely used antibiotics becomes more prevalent (6, 55). In order to reduce mortality from diarrhea in children under 5 years of age to less than 1/1,000 live births by 2025 (56), current antibiotics will have to be complemented by other kinds of treatment, such as host-directed therapies. Given that macrophages and epithelial cells appear to be manipulated by S. flexneri in diametrically opposite ways, developing an adjuvant therapy by targeting a common feature of those two types of host cells may be one way to block bacterial pathogenesis. In this study, we demonstrated that by inhibiting infection-induced acetyl-CoA production in host immune cells, the function of these cells can be restored and energy acquisition by the intracellular pathogen can be limited (Fig. 5). Future studies will involve extensive investigation of more host targets and biological pathways, especially those with unknown functions in bacterial infection. In summary, our study not only sheds new light on the mechanisms underlying S. flexneri-macrophage interactions and *Shigella* pathogenesis but also provides insights into guiding the development of adjuvant therapy for shigellosis treatment.

## MATERIALS AND METHODS

### Reagents.

IRAK1/4-inhibitor I, oxythiamine, and ginsenoside compound K were purchased from Sigma and used at the following concentrations: IRAK1/4-Inh, 0.1 to 10 μM; oxythiamine, 0.01 to 1 mM; and compound K, 1 to 25 μM. Antibiotics in the media were at the following concentrations: 100 μg ml^−1^ ampicillin, 100 μg ml^−1^ gentamicin, and 100 U ml^−1^ penicillin-streptomycin (Pen/Strep; Gibco).

### Mammalian cell culture.

The human monocyte cell line THP-1 was a gift from Jianzhu Chen (Singapore-MIT Alliance for Research and Technology). Cas9-expressing and dCas9-Krab-expressing THP-1 cell lines were constructed in a previous study (31). HEK293FT cells were gifts from Asha Shekaran (Engine Biosciences). THP-1 cells were cultured in RPMI 1640 (HyClone) with 10% fetal bovine serum (FBS; Gibco) and Pen/Strep at 37°C with 5% CO_2_. Phorbol 12-myristate 13-acetate at 50 ng ml^−1^ (PMA; Sigma) was used to differentiate THP-1 cells in tissue culture-treated 96-well plates (Corning) for validation experiments of small-molecule inhibitors. HEK293FT cells were cultured in Dulbecco’s modified Eagle’s medium (DMEM) (HyClone) supplemented with 10% FBS and Pen/Strep at 37°C with 5% CO_2_.

Frozen peripheral blood mononuclear cells were obtained by Ficoll gradient centrifugation of healthy donor leukaphereses (Research Blood Components). Primary human monocytes were isolated by CD14-positive selection (Stemcell Technologies). Monocytes were allowed to mature into macrophages on tissue culture-treated dishes using 50 ng ml^−1^ granulocyte macrophage colony-stimulating factor (GM-CSF) (BioLegend) for 6 days in RPMI 1640 with 10% FBS, 10 mM HEPES, and 1× GlutaMAX (Gibco). Matured macrophages were dissociated with Accutase (Innovative Cell Technologies), counted, distributed in a 96-well plate format, and allowed to adhere overnight in the same medium without GM-CSF. All incubations were performed at 37°C with 5% CO_2_.

### Bacterial strains and growth conditions.

Shigella flexneri serotype 5a strain M90T Δ*virG* pCK100 (P*uhpT*::dsRed), a gift from Cecile Arrieumerlou (Institut Cochin), wild-type S. flexneri M90T, and wild-type S. flexneri serotype 2a strain 2457T were grown on tryptic soy agar (TSA) containing 0.01% Congo red dye at 37°C. Congo red-binding single colonies were inoculated into Luria-Bertani (LB) medium and grown overnight at 37°C with shaking (35, 57–59). The next day, bacteria were diluted 1:100 into 10 ml LB medium and grown to exponential phase for infection. The wild-type S. flexneri M90T and S. flexneri 2457T strains were also grown in tryptic soy broth (TSB) for human primary macrophage infection. The virulence of S. flexneri was verified by spreading cells on Congo red plates at each step and observing retention of the red dye, which indicated that virulence had been retained (60, 61). When necessary, 100 μg ml^−1^ ampicillin was added to the growth medium.

### *In vitro* bacterial infection.

S. flexneri strains were grown to exponential growth phase for host cell infection. To perform CRISPR screens, undifferentiated THP-1 cells were cultured in T225 flasks and infected with the S. flexneri M90T Δ*virG* mutant (S. flexneri Δ*virG*) at an MOI of 10 in complete RPMI 1640 medium for 2 to 3 h. Host cells were treated and maintained with 100 μg ml^−1^ gentamicin to kill extracellular bacteria during the screening process. To validate the function of gene knockdowns in THP-1 cells, undifferentiated host cells were cultured in T25 flasks and infected with S. flexneri Δ*virG* at an MOI of 10 for 2 h. Gene knockdown THP-1 cells were subsequently treated with gentamicin for 2 h, washed twice with 1× phosphate-buffered saline (PBS), and maintained for 24 h after the initial infection and for the rest of the experiments. To validate the functions of small-molecule inhibitors in undifferentiated THP-1 cells, these host cells were pretreated with inhibitors for 1 h in T25 flasks, infected with S. flexneri Δ*virG* at an MOI of 10 for 2 h, and treated with gentamicin for 2 h. After being washed with 1×PBS, the cell culture was maintained with inhibitors for 24 h after the initial infection and for the rest of the experiments. To test IRAK1 and PDHB small-molecule inhibitors in differentiated host cells, PMA-stimulated THP-1 cells and primary human monocyte-derived macrophages (hMDMs) were seeded in 96-well tissue culture plates, pretreated with small-molecule inhibitors for 1 h, and subsequently infected with S. flexneri at an MOI of 1:10. After 2 h of infection, THP-1 cells and hMDMs were treated with gentamicin for 2 h. Subsequently, host cells were washed and maintained with inhibitors for 24 h after the initial infection and for the rest of the experiments. Undifferentiated viable THP-1 cells were counted in a hemocytometer by using trypan blue (Gibco). The cell death of PMA-stimulated THP-1 cells and hMDMs was measured by an LDH assay (TaKaRa).

### Enumeration of intracellular bacteria in infected cells.

At selected time points, 1 ml of *Shigella*-infected undifferentiated THP-1 cells in flasks were centrifuged and washed twice with 1× PBS and then lysed with 50 μl of 1× PBS with 1% Triton X-100. PMA-stimulated THP-1 cells and hMDMs infected with S. flexneri in 96-well plates were lysed with 50 μl of 1× PBS with 1% Triton X-100. Tenfold serial dilutions were performed, followed by plating on LB agar plates, and the plates were incubated at 37°C for 24 h. The number of viable intracellular bacteria was calculated from the counted CFU on the agar plates.

### Pooled genome-wide and secondary CRISPR screens.

The Brunello human CRISPR knockout pooled library was obtained from Addgene (catalog number 73178). The Dolcetto human CRISPRi pooled library was a gift from John Doench (the Broad Institute, also available from Addgene [catalog number 92385]). Both secondary CRISPR knockout and CRISPRi libraries, with 10 sgRNAs per gene, were designed to target the 251 genes scored in primary genome-wide screens (133 of these genes were identified as being involved in S. flexneri infection) and 121 genes from the literature (47 of these genes were found to be involved in S. flexneri infection) (31). One thousand nontargeting sgRNAs were used as controls (31).

### Lentiviral library packaging.

Well-dissociated HEK293FT cells were seeded at a density of 1.4 × 10^7^ cells per flask in a total volume of 35 ml of DMEM 24 h before transfection. Cells were optimal for transfection at 80 to 90% confluence using 7 ml of Opti-MEM, 231 μl of PLUS reagent, 210 μl of Lipofectamine 2000, and a DNA mixture of 18.2 μg of psPAX2 (Addgene catalog number 12260), 11.9 μg of pMD2.G (Addgene catalog number 12259), and 23.8 μg of library plasmid. Flasks were incubated at 37°C with 5% CO_2_ for 4 h. The medium was replaced with 35 ml DMEM with 10% FBS and 1% bovine serum albumin (BSA). Lentivirus was harvested 2 days after the start of transfection and filtered through a 0.45-μm polyethersulfone membrane.

### Lentivirus transduction.

To ensure that only one gene was targeted in each cell, Cas9- and dCas9-Krab-expressing THP-1 cells were transduced with the pooled lentiviral CRISPR knockout and CRISPRi libraries in three biological replicates at an MOI of 0.3. To ensure that each perturbation would be fully represented and reduce spurious effects due to random genome integration in the transduced cell population, screening libraries were prepared with a coverage of >500 cells per sgRNA. Lentiviral spinfection was performed by centrifuging 12-well plates at 1,000 × *g* for 2 h at 33°C with THP-1 cells grown in RPMI 1640 medium with 10% FBS and 8 μg ml^−1^ of Polybrene. Twenty-four hours after lentiviral transduction, cell culture medium was replaced by RPMI 1640 with 10% FBS and 2 μg ml^−1^ of puromycin for selection. Following antibiotic selection, a library coverage of >3,000× was maintained for subsequent screens.

### CRISPR screens.

After puromycin selection, each CRISPR library replicate was split, one used for S. flexneri Δ*virG* infection and one used as a control to verify library representation. Uninfected host cells were pelleted by centrifugation at 600 × *g* for 5 min to extract genomic DNA. Surviving host cells were harvested 2 to 3 weeks after the initial infection and pelleted by centrifugation, with a coverage of >500 cells per sgRNA. The pooled screens were performed as three independent replicates.

### gDNA extraction, barcode amplification, NGS, and analysis.

Genomic DNA (gDNA) from live THP-1 cells was isolated using a modified homemade salt precipitation method as described previously (62). The sgRNA cassette was amplified by PCR and prepared for Illumina sequencing (HiSeq2000) as described previously (32). The sequencing reads were deconvoluted to generate a matrix of read counts, which were then normalized under each condition by the following formula: log_2_(reads per sgRNA/total reads per condition × 10^6^ + 1). The log_2_ fold change of each sgRNA was determined by comparing an infected sample and uninfected samples for each biological replicate. A CRISPR screen analysis tool developed by the Genetic Perturbation Platform (GPP) at the Broad Institute was used to evaluate the rank and statistical significance of genes (https://portals.broadinstitute.org/gpp/public/analysis-tools/crispr-gene-scoring). A hypergeometric distribution was used to calculate the overlap probability of screen hits between CRISPR knockout and CRISPRi screens. Tests were carried out in the R package using the function phyper (q, m, n, k, lower. tail=FALSE, where q is the number of overlap genetic hits, m is the number of genetic hits identified by the CRISPR knockout screen, n is the total number of genes in the library, and k is the number of genetic hits identified by the CRISPRi screen). The GSEA tool (63, 64) was used to perform gene set enrichment analysis based on genetic hits identified by CRISPR screens. Enrichment Map was used for interpretation of the biological processes (65).

### Validation of individual sgRNAs.

For each sgRNA cloning, spacer-encoding sense and antisense oligonucleotides with BsmBI-compatible overhangs were synthesized, annealed, cloned into the lentiGuide-Puro vector (Addgene catalog number 52963), and verified by sequencing (see Table S9 in the supplemental material). Lentivirus was generated in HEK293FT cells using PLUS reagents and Lipofectamine 2000, according to the manufacturer’s instructions. Lentiviral transduction was performed in dCas9-Krab-expressing THP-1 cells to generate individual gene knockdown THP-1 cells. After 11 days of puromycin selection, each individual gene knockdown THP-1 cell was infected with S. flexneri Δ*virG* to validate its phenotype, such as host cell survival and intracellular pathogen growth, as a top genetic hit identified by the CRISPR screens.

10.1128/mBio.02158-21.10 TABLE S9 Genetic hits validated in S. flexneri infection. Download Table S9, DOCX file, 0.02 MB. Copyright © 2021 Lai et al. 2021 Lai et al. https://creativecommons.org/licenses/by/4.0/ This content is distributed under the terms of the Creative Commons Attribution 4.0 International license.

### Cytokine quantification.

Supernatants of cell cultures were collected at the indicated times post-bacterial infection. Cytokine and chemokine levels in S. flexneri-infected supernatants were determined using Bio-plex pro human cytokine 17-plex and IFN-a2 kit (Bio-Rad) according to the manufacturer’s instructions. The results were measured by a Bio-Plex 200 system (Bio-Rad).

### Metabolite profiling.

Metabolite extraction and targeted metabolomics analyses were carried out according to the methods in published reports, with modifications (66). Briefly, cell cultures were harvested at given time points and rapidly quenched, and metabolites were extracted using acetonitrile-methanol-water (2:2:1). After centrifugation, the supernatant was collected and evaporated to dryness in a vacuum evaporator, and the dry extracts were redissolved in 100 μL of 98:2 water-methanol for liquid chromatography-mass spectrometry (LC-MS) analysis.

The targeted LC-MS/MS analysis was performed with the Agilent 1290 ultrahigh-pressure liquid chromatography system coupled to a 6490 triple-quadrupole mass spectrometer equipped with a dual-spray electrospray ionization source with Jet Stream (Agilent Technologies, Santa Clara, CA). Chromatographic separation of metabolites in central carbon metabolism was achieved by using a Phenomenex (Torrance, CA) Rezex ROA-organic acid H^+^ (8%) column (2.1 by 100 mm, 3 μm), and the compounds were eluted at 40°C with an isocratic flow rate of 0.3 mL min^−1^ of 0.1% formic acid in water. Compounds were quantified in multiple-reaction-monitoring (MRM) mode. Electrospray ionization was performed in both the positive and negative ion modes with the following source parameters: a drying gas temperature of 300°C with a flow of 10 L min^−1^, a nebulizer gas pressure of 40 lb/in^2^, a sheath gas temperature of 350°C with a flow of 11 L min^−1^, a nozzle voltage of 500 V, and capillary voltages of 4,000 V and 3,000 V for the positive and negative modes, respectively. Data acquisition and processing were performed using MassHunter software (Agilent Technologies, USA), and cell counts were normalized to correct variations in sample preparation.

### Imaging.

To visualize intracellular RFP-reporter S. flexneri M90T Δ*virG* (P*uhpT*::dsRed), infected THP-1 cells were directly observed under a confocal fluorescence microscope (Zeiss LSM 700).

### Quantification and statistical analysis.

Excel was used for all statistical analyses. All of the statistical details of the experiments can be found in the corresponding figure legends.

Tables S1 to S7 can be found at https://doi.org/10.17632/xn3vv2cdnk.1.
