# Supplementary material for: High-Throughput CRISPR Screens To Dissect Macrophage-Shigella Interactions
Source: mBio. 2021 Dec 21;12(6):e02158-21. doi: 10.1128/mBio.02158-21 (PMC8689513; doi:10.1128/mBio.02158-21)
Supplement: TABLE S9 [file mbio.02158-21-st009.docx]

**Table S9. Genetic hits validated in S. flexneri ∆virG infection.**

| **Bacterial infection** | **Pathways** | **Screen hits** | **sgRNA target sequence** |
| --- | --- | --- | --- |
| *S. flexneri* M90T infection | Toll-like receptors cascades | TRAF6 | GCGGCCAGCGAAGGTGGCGA |
|  |  | IRAK1 | GGACCTGCCGGGGCCTCTCA |
|  |  | MYD88 | CCTCCTGCAGCCATGGCGGG |
|  | Pyruvate metabolism | PDHB | GGGAGGTAGGCAGCAGCGCG |
|  |  | DLAT | GGGGGGTTGGTGGCACTATG |
|  |  | CS | GCCGCGCCGACGGGTTGACA |
|  | Type I IFN signaling pathway | TYK2 | TCAAGCGCAGCCAGTCCCCG |
|  | Transcriptional regulation of TP53 | TP73 | AAGGGGACGCAGCGAAACCG |
|  | Apoptosis Modulation and Signaling | TNFRSF1A | CTGGACTGAGGCTCCAGTTC |
|  | Cytosolic immune receptor | ALPK1 | CTGCTCAGAGTTAGATTTGC |
|  | Inflammasome | NLRP3 | TCTGGGTAAGTCCAGCTCCG |
|  |  | GSDMD | TCCAGCTCCTGCTCGCCGGA |
|  | Unknown | PHIP | TGAATGGTGGAGCCGAAGCT |
|  |  | PHF6 | TCCAGCAGTGCCTGAGAGCG |
|  |  | TRERF1 | GGAGAGTTTGGAGTTGCTTG |
| Non-targeting control |  | NC80 | ACATGTGGCTCCGCCCACAG |
|  |  | NC135 | AGATCTGCTCCATGTCACCA |
